# Targeting SLITRK4 Restrains Proliferation and Liver Metastasis in Colorectal Cancer via Regulating PI3K/AKT/NFκB Pathway and Tumor‐Associated Macrophage

**DOI:** 10.1002/advs.202400367

**Published:** 2024-11-05

**Authors:** Xiaojiao Sun, Junling Zhang, Bingqi Dong, Qingqing Xiong, Xin Wang, Yanlun Gu, Zhiqi Wang, Huiyu Liu, Jixin Zhang, Xu He, Hongjin Liu, Yi Zhong, Chuxiao Yi, Xiaowei Chi, Zhenming Liu, Xiaocong Pang, Yimin Cui

**Affiliations:** ^1^ State Key Laboratory of Natural and Biomimetic Drugs School of Pharmaceutical Sciences Peking University Beijing 100191 China; ^2^ Department of General Surgery Peking University First Hospital Xishiku Street, Beijing Xicheng 100034 China; ^3^ Department of Hepatobiliary Cancer Liver Cancer Center Tianjin Medical University Cancer Institute Tianjin 300060 China; ^4^ Department of Pharmacy Peking University First Hospital Xishiku Street, Beijing Xicheng 100034 China; ^5^ Institute of Clinical Pharmacology Peking University Xueyuan Road 38, Beijing Haidian 100191 China; ^6^ Department of Pathology Peking University First Hospital Xishiku Street, Beijing Xicheng 100034 China

**Keywords:** colorectal cancer, liver metastasis, macrophage, nanoparticles, SLITRK4

## Abstract

Liver metastasis is the major cause of death in colorectal cancer (CRC) due to the lack of effective treatment. To explore novel drivers of CRC liver metastasis, the transcriptomes of primary paracancerous, colorectal tumors and metastases from human patients are profiled. It is found that SLIT‐ and NTRK‐like family member 4 (SLITRK4) is the top upregulated gene in liver metastases and is associated with worse overall survival of CRC patients. Multiple in vitro and in vivo models suggested SLITRK4 promoted CRC tumorigenesis, invasion, migration, and angiogenesis, and inhibition of it restrained CRC tumor growth and liver metastasis with a more profound effect on the tumor microenvironment (TME). Mechanistically, SLITRK4 overexpression significantly activated the PI3K/AKT/NFκB pathway, regulated extracellular matrix organization, and multiple cytokines expression. Furthermore, the results from coculture models and single‐cell RNA sequencing analyses suggested SLITRK4 promoted tumor‐associated macrophages (TAMs) infiltration and polarization. In addition, macrophage depletion significantly inhibited SLITRK4‐induced liver metastasis in CRC. Finally, pharmacological inhibition of SLITRK4 by using lipid‐polymer hybrid nanoparticles (NPs) for systemic siRNA delivery can effectively inhibit CRC liver metastasis. Taken together, these results pinpoint that SLITRK4 regulates CRC tumorigenesis and liver metastasis, and siRNA delivering NPs agents validate the therapeutic potential of targeting SLITRK4 in CRC.

## Graphical abstract

This study indicates that SLITRK4 plays a newly pro‐oncogenic role in exacerbating colorectal cancer (CRC) tumorigenesis and metastasis. Inhibition of SLITRK4 significantly suppresses CRC liver metastasis by modulating cell‐matrix adhesion, reducing angiogenesis and tumor‐associated macrophage infiltration in the tumor microenvironment. In addition, lipid‐polymer hybrid nanoparticles for siSLITRK4 delivery can be a promising strategy for CRC liver metastasis therapy.

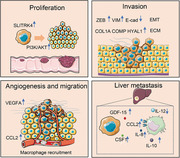

## 1 Introduction

Colorectal cancer (CRC) is the third most prevalent neoplasm (10%) and the second leading cause of cancer‐related mortality (9.4%) all over the world.^[^ ^1^ ^]^ Beyond half of CRC, patients eventually suffer from liver metastasis during tumor development, which significantly affects the 5‐year survival rate. The postoperative 5‐year survival rate of CRC patients with local tumors is over 90%, and once they develop liver metastasis, the 5‐year survival rate of CRC patients with liver metastasis is only 9% without curative resection.^[^ ^2^, ^3^ ^]^ Therefore, liver metastasis is thought to be a great threat to the poor prognosis of CRC. It is urgent to further explore the mechanisms of CRC liver metastasis and identify novel targets to enhance the overall survival of CRC patients.

The neurotrophic tyrosine receptor kinase (NTRK) gene has a dual function in promoting nervous system development and carcinogenesis.^[^ ^4^ ^]^ In the 1950s, researchers discovered that some neurotrophic factors such as NT3, NT4, and BDNF were involved in the development of the nervous system, and afterward NTRK was discovered as a receptor for these neurotrophic factors.^[^ ^4^ ^]^ In 1982, NTRK was first discovered their function as an oncogene in CRC. Subsequently, NTRK fusion mutations have also been found in multiple tumors such as thyroid cancer and fibrous astrocytoma.^[^ ^5^ ^]^ When the NTRK gene fuses with other genes, the abnormal Trk fusion protein can remain ligand independent and continuously activate downstream MAPK, PI3K, and PKC signaling pathways, promoting tumor cell proliferation and differentiation.^[^ ^5^ ^]^

Herein, by comparing CRC samples from primary tumors with liver metastases, we found that SLIT‐ and NTRK‐like family member 4 (SLITRK4) had higher expression in liver metastases and was related to poor clinical prognosis. SLITRK encodes a family of transmembrane proteins, including SLITRK 1–6, which contain leucine‐rich repeat (LRR) domains^[^ ^6^ ^]^ like the extracellular region of NTRK. All SLITRK 1–6 are highly expressed in the nervous system and mediate basic neuronal processes, including neurite migration, dendritic branching, neuronal survival, and synapse formation.^[^ ^7^ ^]^ Similar to NTRK, previous studies on SLITRK have mainly focused on its function in nervous system diseases. In our study, we found that SLITRK4 is a relatively specific risk factor for gastrointestinal cancer. Recently, Zhou et al., found that the interaction between SLTRK4 and canopy FGF signaling regulator 3 (CNPY3) could enhance the TrkB‐related signaling pathway in gastric cancer metastasis.^[^ ^8^ ^]^ Metastasis is a multistep process mediated by key genes regulating the complex cell‐extrinsic interaction with surrounding stromal cells, but the role of SLITRK4 in the tumor microenvironment (TME) is still unknown.

Recent advances have shown that the abundance of tumor‐associated macrophages (TAMs) is strongly associated with poor clinical outcomes in multiple solid cancers, including CRC, hepatocellular carcinoma, gastric cancer, prostate cancer, and breast cancer.^[^ ^9^, ^10^, ^11^, ^12^, ^13^, ^14^ ^]^ In CRC, the plasticity and heterogeneity of macrophage were influenced by the TME, such as high levels of IL‐6 stimulating TAMs toward an anti‐inflammatory phenotype.^[^ ^15^ ^]^ Recently, single‐cell RNA sequencing of matched CRC, adjacent colon, and liver metastasis samples indicated that the TME in the liver metastatic site underwent remarkable spatial reprogramming of MRC1+ CCL18+ macrophages and contributed to the formation of a suppressive immune microenvironment.^[^ ^14^ ^]^ Macrophages are sufficiently plastic to integrate multiple microenvironment signals and recent advances have provided a framework for macrophage polarization.^[^ ^16^, ^17^ ^]^ However, the mechanisms of TAMs recruitment and polarization in CRC liver metastases are poorly understood.

Herein, we identified SLITRK4 as a driver of CRC liver metastasis and a promising target for CRC with liver metastasis. A series of tumorigenic function assays suggested that it participated in tumor cell proliferation, migration, invasion, angiogenesis, and metastasis. In addition, overexpression of SLITRK4 boosted the transcription of cytokines related to macrophage polarization, such as IL‐8, CCL2, GDF15, and CSF‐1 by activating the PI3K/AKT/NFκB pathway, and enhanced the infiltration of CD206^+^CD11b^+^TAMs in CRC liver metastases. Furthermore, macrophage depletion significantly impeded liver metastasis in CRC. In addition, single‐cell RNA sequencing suggested that knockdown of SLITRK4 could improve the suppressive immune microenvironment by promoting CD8^+^ T cell recruitment. Finally, a small interfering RNA targeting SLITRK4 encapsulated into a lipid‐polymer hybrid nanoparticles platform (siSLITRK4‐NPs) significantly suppressed CRC tumor growth and liver metastasis. Taken together, our findings suggest that targeting SLITRK4 is a potent treatment strategy for CRC liver metastasis.

## 2 Results

### 2.1 SLITRK4 Expression was Correlated with CRC Liver Metastasis

**Figure 1:**
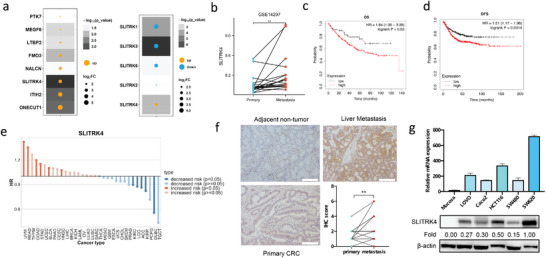
SLITRK4 was highly expressed in CRC liver metastasis. a) mRNA abundances of differentially expressed genes in the internal cohort. The background shading represents the P value, and the size and color of the dot represent the log2FC of the liver metastasis over the primary tumor. b) mRNA expression of SLITRK4 in GSE14297, including paired 18 primary colorectal cancers and its matched liver metastases (paired *t*‐test). c,d) Association of SLITRK4 expression with overall survival and disease‐free survival of CRC patients. e) The mortality risk assessment of SLITRK4 in cancer types via the TISCH database based on the TCGA pan‐cancer datasets. f) Representative images of SLITRK4 protein expression in paired 21 CRC liver metastases and its matched primary tumors by IHC. Scale bars, 500 µm. The protein levels of SLITRK4 were assessed using Allred's score (paired *t*‐test). Allred's score is the sum of two numbers. The first number represents the percentage of positive tumor cells (0 represents 0, 1 represents<1%, 2 represents 1%–10%, 3 represents 11%–33%, 4 represents 34%–66%, and 5 represents 67%–100%), and the other number represents the staining intensity (0 represents none, 1 represents weak, 2 represents moderate, and 3 represents strong). g) The RNA and protein expression levels of SLITRK4 in five CRC cell lines and normal mucosal cells were examined. All histogram chart data are presented as the mean ± SD. ^*^ *p* < 0.05, ^**^ *p* < 0.01, ^***^ *p* < 0.001, ^****^ *p* < 0.0001.

We first analyzed differential mRNA expression based on RNA‐seq data from 4 paracancerous, 3 primary CRC, and 6 liver metastasis samples, revealing that SLITRK4 was the most significantly upregulated gene in CRC liver metastasis (**Figure** **1**a) compared with primary CRC. Interestingly, among SLITRK 1–6, only SLITRK4 was specifically upregulated in liver metastasis samples. Consistently, the SLITRK4 expression level was also significantly enhanced in liver metastasis tissues compared with their matched primary CRC tissues in the GSE14297 CRC cohort (Figure 1b). In addition, Kaplan–Meier analyses of the COAD‐TCGA cohort indicated that CRC patients with high SLITRK4 expression had remarkably worse overall survival (OS) and disease‐free survival (DFS) than those with low SLITRK4 expression (Figure 1c,d). Based on the results of univariate Cox regression, multivariate Cox regression, and nomogram analysis, SLITRK4 was significantly associated with CRC patient's OS, which suggested SLITRK4 could be considered as an independent prognostic factor (Figure S1, Supporting Information). We further investigated the role of SLITRK4 in other cancer types, which suggested that overexpression of SLITRK4 significantly increased the mortality risk of gastrointestinal cancer, including CRC and gastric cancer (Figure 1e). We next evaluated the expression of SLITRK4 in CRC cell lines and human tissue samples. Immunohistochemistry (IHC) was conducted to confirm the overexpression of SLITRK4 protein in matched primary CRC and liver metastasis samples (Figure 1f). SLITRK4 expression was dramatically increased in all five CRC cell lines (LOVO, Caco2, SW480, SW620, and HCT116) compared with normal mucosa cells at both the mRNA and protein levels (Figure 1g). We also noted that SLITRK4 was elevated in SW620 cell lines compared with SW480 cell lines (Figure 1g), a pair of cells isolated from the metastatic and primary sites of a single patient, respectively, which represent different metastatic abilities. This result also hinted at the relationship between SLITRK4 and metastasis. Therefore, our results suggested that SLITRK4 was upregulated in CRC with liver metastasis compared with primary CRC tissue.

### 2.2 SLITRK4 Promoted Tumorigenesis and Metastasis in Multiple CRC Models

**Figure 2:**
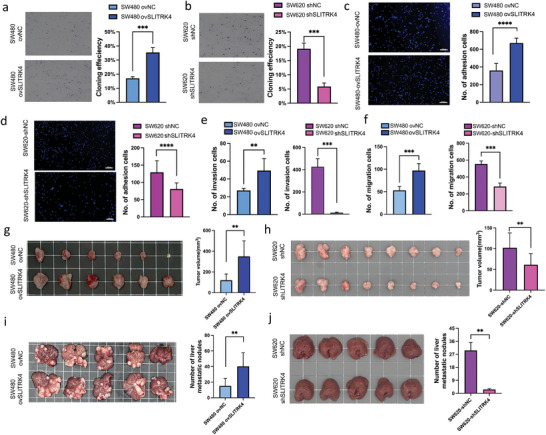
SLITRK4 promoted CRC cell growth and metastasis in vitro and in vivo. a,b) The effect of SLITRK4 on CRC cell proliferation was tested by colony formation assay (n = 3 biological replicates). c,d) Cell adhesion assay was used to compare SLITRK4 knockdown or overexpression with control cell lines. (n = 3 biological replicates). Scale bar, 200 µm. e,f) The effect of SLITRK4 on CRC cell invasion and migration was detected by transwell assay. (n = 3 biological replicates). g) Representative images and tumor volumes derived from SW480 cells with and without SLITRK4 overexpression (n = 6 mice in each group). h) Representative images and tumor volume derived from SW620 cells withand without SLITRK4 knockdown(n = 8 mice in each group). i) Representative images of liver metastatic tissue and the number of liver metastatic nodules from SW480 cells with and without SLITRK4 overexpression (n = 5 mice in each group). j) Representative images of liver metastatic tissue and the number of liver metastatic nodules from SW620 cells with and without SLITRK4 knockdown (n = 5 mice in each group). All histogram chart data are presented as the mean ± SD. Statistical analyses were performed by Graphpad Prism 10.1.2 using the two‐tailed Student's *t*‐test to detect differences between the groups. ^*^ *p* < 0.05, ^**^ *p* < 0.01, ^***^ *p* < 0.001, ^****^ *p* < 0.0001.

We next investigated the role of SLITRK4 in human CRC cell lines. Knockdown of SLITRK4 suppressed cell proliferation and colony formation of SW620 cells, reciprocally, overexpression of SLITRK4 promoted cell proliferation significantly (**Figure** **2**a,b; Figure S2a,b, Supporting Information). We further determined the function of SLITRK4 in the prometastatic abilities of CRC cells. Cell adhesion assays also showed that the adhesion ability of SW620 cells and HCT116 cells was impaired after SLITRK4 knockdown, whereas overexpression of SLITRK4 resulted in increased adhesion ability (Figure 2c,d; Figure S2c, Supporting Information). Transwell assays indicated that SLITRK4 knockdown significantly reduced CRC cell invasion and migration, whereas the cell migration and invasion capacities of CRC cells were evidently enhanced upon SLITRK4 overexpression (Figure 2e,f; Figure S2c, Supporting Information). To confirm the roles of SLITRK4 in vivo, we utilized subcutaneous xenotransplantation models to evaluate whether SLITRK4 contributed to CRC progression. Consistent with the observation in vitro, when SLITRK4‐knockdown SW620 cells were implanted, the tumor growth rate was slower than that of the control in vivo, otherwise, SLITRK4 overexpression resulted in significantly higher proliferation (Figure 2g,h). SLITRK4 knockdown and overexpression assays in mouse liver metastasis models further validated the metastasis‐promoting effect of SLITRK4 in vivo (Figure 2i,j). Epithelial‐mesenchymal transition (EMT) has great significance in the CRC cell metastatic process.^[^ ^18^ ^]^ We found that SLITRK4 significantly regulated the transcription factor Zeb‐mediated EMT process and mediated markers (E‐cadherin and Vimentin) (Figure S2d,e, Supporting Information). Taken together, the results suggested that SLITRK4 promoted CRC cell growth, motility, invasiveness, and liver metastasis.

### 2.3 Transcriptome Profiling Suggested the Functional Role of SLITRK4 in Tumor Growth and Metastasis

**Figure 3:**
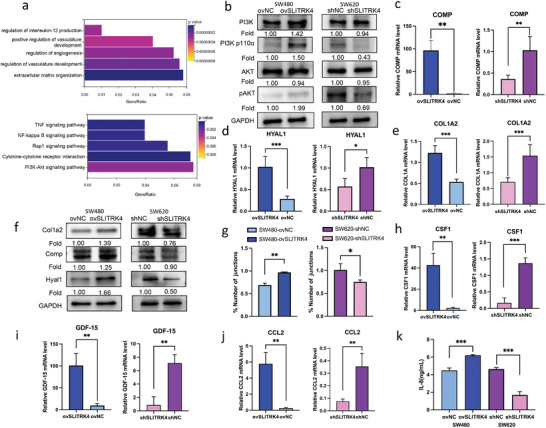
Transcriptome analysis and validation for identifying SLITRK4 targets involved in CRC. a) RNA‐seq analysis in SLITRK4‐overexpressing and control SW480 cells. GO (top) and KEGG (bottom) enrichment of RNA‐seq differentially expressed genes. b) The effect of SLITRK4 on the expression of PI3K/AKT‐related proteins was evaluated by Western blot. Fold change was computed by comparison with ovNC or shNC group. c,d,e,f) The effect of SLITRK4 on the expression of ECM‐related genes was evaluated by qRT‐PCR and Western blot. Fold change was computed by comparison with ovNC or shNC group. g) SW480‐conditioned medium with SLITRK4 overexpression and SW620‐conditioned medium with SLITRK4 knockdown regulated the tube formation of HUVECs. h,i,j,k) The effect of SLITRK4 on the expression of cytokines (GDF‐15, CCL2, CSF1) was measured by qRT‐PCR. (k) The effect of SLITRK4 on the expression of the cytokine IL‐8 was detected by ELISA. All histogram chart data are presented as the mean ± SD. Statistical analyses were performed by Graphpad Prism 10.1.2 using the two‐tailed Student's *t*‐test to detect differences between the groups. n = 3 biological replicates.^*^ *p* < 0.05, ^**^ *p* < 0.01, ^***^ *p* < 0.001, ^****^ *p* < 0.0001.

**Figure 4:**
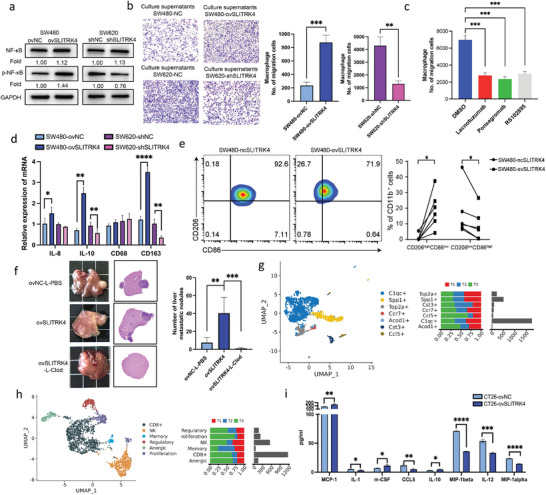
SLITRK4 expression in CRC cells promoted TAM recruitment and polarization in vitro and in vivo. a) The effect of SLITRK4 on the expression of NF‐κB‐related proteins was evaluated by Western blot. Fold change was computed by comparison with ovNC or shNC group. b) The effect of SLITRK4 on macrophage migration was detected by transwell assay. (n = 3, Magnification ×50) c) The effect of CCR2 inhibitor RS102895, GDF15 antibody inhibitor Ponsegromab, and CSF‐1 neutralizing antibody Lacnotuzumab on SLITRK4 overexpression‐induced macrophage migration was detected by transwell assay. (n = 3) d) Macrophage polarization‐related markers were tested by qRT‐PCR in THP1 cells cocultured with supernatants from SW480 and SW620 cells with overexpression or knockdown of SLITRK4. (n = 3) e) Representative flow cytometry analysis and scatterplots of the proportion of CD206^high^CD86^low^ and CD206^low^CD86^high^ in the medium of SW480‐ncSLITRK4 and SW480‐ovSLITRK4 treated human monocyte‐derived macrophages at day 2. (n = 6). f) The effect of macrophage on the SLITRK4‐induced liver metastasis. Chemical method of clodronate liposome (L‐Clod) to deplete macrophages in CT26‐ovSLITRK4 liver metastasis lesions. Representative liver metastases and HE image of mouse liver with colorectal cancer metastases (n = 4 mice in each group). Macrophage depletion significantly inhibited SLITRK4‐induced liver metastasis. g) UMAP plot shows TAM subtypes and Cell composition distribution for groups. control group (T1), SLITRK4 overexpression group (T2), SLITRK4 knockdown group (T3). h) UMAP plot shows T‐cell subtypes and Cell composition distribution for groups. control group (T1), SLITRK4 overexpression group (T2), SLITRK4 knockdown group (T3). i) Typical cytokine levels were determined by Luminex assays in the plasma of the CT26 xenograft model (n = 4 mice in each group). All histogram chart data are presented as the mean ± SD. Statistical analyses were performed by Graphpad Prism 10.1.2 using the two‐tailed Student's *t*‐test to detect differences between the groups. ^*^ *p* < 0.05, ^**^ *p* < 0.01, ^***^ *p* < 0.001, ^****^ *p* < 0.0001.

To clarify the potential mechanism underlying the oncogenic role of SLITRK4, we performed RNA‐seq in SLITRK4‐overexpressing and control SW480 cells. A total of 648 upregulated and 1468 downregulated genes were identified by DESeq in SW480 cells with SLITRK4 overexpression compared with control cells (Figure S2f, Supporting Information). KEGG enrichment suggested that PI3K/AKT signaling was the most significantly regulated pathway by SLITRK4 (**Figure** **3**a), which plays an essential role in cell proliferation and survival.^[^ ^19^ ^]^ Knockdown of SLITRK4 downregulated the phosphorylation of PI3K and AKT (Figure 3b). GO biological pathway (BP) enrichment analysis indicated that “extracellular matrix organization”, “regulation of vasculature”, “regulation of angiogenesis” and “regulation of cytokine production” were significantly enriched in the SLITRK4 overexpression group (Figure 3a). In addition, KEGG enrichment also suggested that cytokine–cytokine receptor interactions were enriched (Figure 3a). We thus validated the effect of SLITRK4 on the expression of extracellular matrix genes, regulation of angiogenesis, and cytokine expression level by quantitative PCR. We noticed that the mRNA levels of most extracellular matrix genes were significantly upregulated upon SLITRK4 overexpression, reciprocally, knockdown of SLITRK4 reduced their expression at both the mRNA and protein levels (Figure 3c,d,e,f; Figure S2 g, Supporting Information). In particular, high expression of COL1A2 and cartilage oligomeric matrix protein (COMP) was associated with worse overall survival of CRC patients (Figure S2 h, Supporting Information). Angiogenesis exerts a key role in the metastasis of CRC cells. The coculture model of SLITRK4 knockdown or overexpression CRC cells with HUVECs indicated that SLITRK4 improved the function of HUVECs, as evidenced by the increased numbers of junctions formed by HUVECs (Figure 3g). In addition, we found that SLITRK4 knockdown remarkably inhibited the secretion of VEGFA, reciprocally, SLITRK4 overexpression promoted VEGFA secretion (Figure S2i, Supporting Information). Considering the potential regulation of cytokine production, we further evaluated the correlation of SLITRK4 with tumor‐infiltrating lymphocytes via Tumor Immune Estimation Resource (TIMER). As a result, SLITRK4 expression was positively correlated with the level of macrophage infiltration in colon adenocarcinoma (COAD) (Figure S2j, Supporting Information). The cytokines C‐C motif chemokine 2 (CCL2), macrophage colony‐stimulating factor 1 (CSF1), and growth differentiation factor 15 (GDF15) produced by tumor cells are the main regulators of macrophage infiltration and survival.^[^ ^20^ ^]^ qPCR detection (Figure 3h–j) suggested that the mRNA expression of CCL2, CSF1, and GDF15 was dramatically decreased upon SLITRK4 knockdown, whereas SLITRK4 overexpression promoted their production. The chemokine CCL8 (also known as IL‐8) exerts a tumor‐promoting effect in cancers by enhancing the infiltration of TAMs. ELISA analysis also suggested that SLITRK4 strongly regulated the production of IL‐8 (Figure 3k). Therefore, these findings suggest that SLITRK4 may influence macrophage infiltration. KEGG enrichment also suggested that NF‐κB signaling was a remarkably regulated pathway by SLITRK4 (Figure 3a). PI3K/AKT and its downstream NF‐κB signaling are the typical regulators of the secretion of cytokines. SLITRK4 remarkably regulated the phosphorylation of PI3K, AKT, and NFκB (Figures 3b and **4**a). In addition, PI3K inhibitor LY294002 effectively inhibited SLITRK4 overexpression and induced the secretion of cytokines, such as CCL2, CSF1, GDF15 and IL‐8, and NF‐κB inhibitor BAY11‐7082 had similar regulation on CCL2, CSF1 and IL‐8 (Figure S3a, Supporting Information). Therefore, SLITRK4 overexpression significantly activated PI3K/AKT/NFκB signaling and further promoted TAMs related cytokines secretion.

### 2.4 SLITRK4 Induces Macrophage Recruitment and Polarization

Considering that SLITRK4 induced cytokines, including CCL2, CSF1, GDF15, and IL‐8, can elevate the levels of protumorigenic macrophages, we evaluated the effect of SLITRK4 on macrophage infiltration and polarization. When primary human peripheral blood‐derived monocytes (PBMCs) cells in suspension culture were cultured with supernatant from SW480 and SW620 with SLITRK4 knockdown or overexpression for 48 h, the migration of macrophages cultured with supernatant from SW480 cells overexpressing SLITRK4 significantly increased, whereas SLITRK4 knockdown resulted in decreased migration (**Figure** **4**b). Meanwhile, SW480 cells overexpressing SLITRK4 induced macrophage migration was remarkably inhibited by CCR2 inhibitor RS102895, GDF15 antibody inhibitor Ponsegromab and CSF‐1 neutralizing antibody Lacnotuzumab (Figure 4c; Figure S3b, Supporting Information). Therefore, SLITRK4 overexpressed induced cytokines promoted macrophage infiltration. Furthermore, cellular supernatant from SW480 cells overexpressing SLITRK4 led to obvious upregulation on the mRNA levels of the TAMs marker CD163 and the characteristic cytokine IL‐10 (Figure 4d), which indicated that SLITRK4 induced a significant bias toward CD163^+^ phenotype in THP1 cells. In contrast, the knockdown of SLITRK4 can block the transition to the CD163^+^ phenotype. The results of flow cytometry also suggested that PBMCs cells cultured with supernatant from SW480 overexpressing SLITRK4 also led to a significant bias toward CD206^high^CD86^low^ (Figure 4e). We further evaluate whether RS102895, Ponsegromab, or Lacnotuzumab SLITRK4 overexpression induced macrophage polarization. As a result from Figure S3c (Supporting Information), RS102895 and Ponsegromab had a strong effect on the mRNA levels of the TAMs marker CD163. Therefore, blocking CCR2 and GSF‐1 receptors showed strong regulation of both macrophage infiltration and polarization.

Next, to test SLITRK4‐induced liver metastasis associated with macrophage, we used the classical chemical method of clodronate liposome (L‐Clod) to deplete macrophages in CT26‐ovSLITRK4 liver metastasis lesions. Interestingly, macrophage depletion significantly inhibited SLITRK4‐induced liver metastasis (Figure 4f). Therefore, SLITRK4 plays a crucial role in the impact of TAMs recruitment and polarization, and TAMs further promote SLITRK4‐induced liver metastasis.

To evaluate the impact of SLITRK4 overexpression on global changes in immune cells from the TME, we performed single‐cell RNA sequencing (scRNA‐seq) in CT26 CRC liver tumors by injecting with CT26, CT‐26‐shRNA, and CT26‐ovSLITRK4 cells into the spleens of BALB/c mice. After 2 weeks, the mice were sacrificed, and liver metastases were detected. Assessment of gene expression in tumor infiltrating cells by scRNA‐seq confirmed that SLITRK4 caused significant changes in the TAMs and T‐cell populations in the tumor (Figure S4a,b,c, Supporting Information; Figure 4g,h). Macrophages were identified by showing high expression levels of CD68. The unbiased analysis identified seven TAMs populations with distinct gene expression profiles within CT26 tumors, and among them, C1qc+ TAMs accounted for the largest proportion (Figure 4g; Figure S4b, Supporting Information). C1qc+ TAMs were reported to mediate dysfunctional T cell composition via cytokine/chemokine signaling.^[^ ^21^ ^]^ The Ccr7+ TAMs and Ccl5+ TAMs showed a significant reduction in the SLITRK4 overexpression group (T2) compared to the control group (T1), whereas their abundance levels were enhanced in the SLITRK4 knockdown group (T3). Typical pro‐inflammatory cytokines, such as CCL9 and CCL5, were significantly upregulated in T3 (Figure 4g; Figure S4d, Supporting Information). Next, we evaluated the effect of SLITRK4 on the composition of T‐cell infiltrates. Analysis of the T‐cell infiltrates showed the highest abundance of CD8^+^T cells (Figure 4h). In addition, a significant increase in the proportion of CD8^+^T and NK cells was observed upon STLITRK4 knockdown. Typical cytokine levels were determined by Luminex assays in the CT26 xenograft model. Overexpression of SLITRK4 significantly improved the production of MCP‐1, m‐CSF, and IL‐10 and reduced the production of IL‐1, CCL5, MIP‐1alpha, MIP‐1beta, and IL‐12 in plasma (Figure 4i). Taken together, SLITRK4 plays a key role in the promotion of macrophage recruitment and polarization, and overexpression of SLITRK4 generally leads to a suppressive immune microenvironment.

### 2.5 Targeting SLITRK4 by Systemic Delivery of siRNA Using Lipid‐Polymer Hybrid Nanoparticles (NPs) for CRC Liver Metastasis Therapy

**Figure 5:**
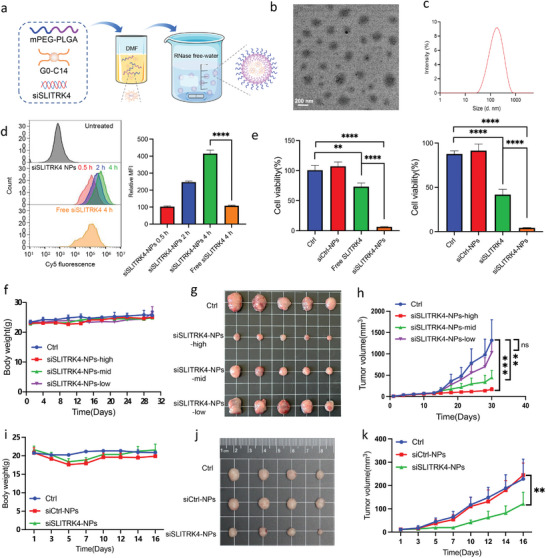
Targeting SLITRK4 by systemic delivery of siRNA using lipid‐polymer hybrid NPs for CRC liver metastasis therapy. a) Schematic illustration for the preparation of siSLTRK4‐encapsulated lipid‐polymer hybrid NPs. b) TEM image of siSLTRK4‐NPs. c) The size distribution histogram of siSLTRK4‐NPs detected by DLS. d) The mean fluorescence intensity (MFI) of SW620 cells following treatment with cy5‐labeled siSLITRK4 or siSLITRK4‐NPs was determined by flow cytometry. n = 3 biological replicates. e) The anti‐proliferation and toxicity of siSLITRK4‐NPs and siCtrl‐NPs were evaluated in SW620 and HCT116 cells by CCK‐8 assays. n = 3 biological replicates. f) The body weight change curves of the mice during the course of treatment in the SW620 xenograft model (n = 5 mice in each group). g) Representative image of tumor xenografts harvested 30 days after different treatments. h) Tumor growth curves of SW620 xenografts after different treatments. i) The body weight change curves of the mice during the course of treatment (n = 4 mice in each group) in the HCT116 xenograft model. j) Representative image of tumor xenografts harvested 16 days after different treatments. k) Tumor growth curves of HCT116 xenografts after different treatments. All histogram and line chart data are presented as the mean ± SD. Statistical analyses were performed by Graphpad Prism 10.1.2 using the two‐tailed Student's *t*‐test to detect differences between the groups.^*^ *p* < 0.05, ^**^ *p* < 0.01, ^***^ *p* < 0.001, ^****^ *p* < 0.0001.

Since no crystal structure is available, it is difficult to find molecules that directly target SLITRK4. Recently, we successfully developed a lipid‐polymer hybrid nanoparticle platform for the systemic delivery of siRNA to tumors. Therefore, we used the NPs platform for delivering siSLITRK4 for CRC liver metastasis therapy. The siSLITRK4‐NPs were self‐assembled from mPEG‐PLGA copolymers, in which the complexes of siRNA with a cationic lipid–like material G0‐C14 are loaded (**Figure** **5**a). The morphology and hydrodynamic diameter of siSLITRK4‐NPs were evaluated by transmission electron microscopy (TEM) and dynamic light scattering (DLS), respectively (Figure 5b,c; Figure S5, Supporting Information). The hydrodynamic size was 141 nm with a polydispersity index (PDI) of 0.251 (Figure 5c). Next, we tested the intracellular uptake of fluorescence‐labeled siSLITRK4‐NPs, in which siSLITRK4 was replaced by cy5‐labeled siSLITRK4, in SW620 cells by flow cytometry. SW620 cells were incubated with cy5‐labeled siSLITRK4‐NPs for 0.5, 2, or 4 h. As shown in Figure 5d, the mean fluorescence intensity (MFI) of cy5‐labeled siSLITRK4 was increased as the incubation time was prolonged. In addition, the MFI of SW620 cells treated with siSLITRK4‐NPs for 4 h was significantly higher than that of cells treated with free siSLITRK4, suggesting that the NPs platform could efficiently deliver siSLITRK4 into tumor cells. Next, we determined whether siRNA‐loaded NPs could improve cell growth inhibition compared with naked siRNA. Figure 5e indicates that the viability of both SW620 and HCT116 cells treated with siSLITRK4‐NPs for 48 h was significantly reduced compared with that of cells treated with free siRNA. Meanwhile, siCtrl‐NPs had no influence on the cell viability of the two cells, which indicated the good biocompatibility of the NPs platform.

Next, the in vivo antitumor efficacy of siSLITRK4‐NPs was evaluated. As shown in Figure 5f, SW620 tumor‐bearing BALB/c nude mouse models were generated and then administered with PBS, siCtrl‐NPs, or siSLITRK4‐NPs via tail vein injection at a fixed siRNA dose of 0.5, 1.0, 2.0 nmol per mouse on days 1, 3, 6, and 7. Consistent with the in vitro experiments, siSLITRK4‐NPs remarkably restrained SW620 xenografts growth in median and high dose administration groups compared with the control group (Figure 5g,h). No obvious changes in body weight were observed during the course of therapy (Figure 5f). HCT116 tumor‐bearing BALB/c nude mouse models were also used for the evaluation of the antitumor efficacy of siSLITRK4‐NPs at 1.0 nmol per mouse (Figure 5i,j,k). For the mice in the two control groups, the tumor volume was more than 2 fold larger than those in the siSLITRK4 NPs group (Figure 5j,k). In addition, IHC of SLITRK4, Ki‐67, and CD31 confirmed the reduction of SLITRK4 expression and inhibition of growth rate and angiogenesis in the siSLITRK4‐NPs group compared with the control NPs group (Figure S5d–h, Supporting Information).

**Figure 6:**
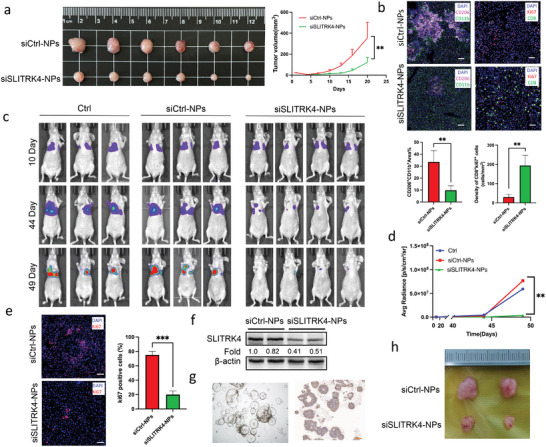
Therapeutic efficacy of siSLITRK4‐NPs in a CRC liver metastasis and subcutaneous tumor models. a) Representative image of CT26 tumor xenografts harvested 20 days after different treatments (n = 6 mice in each group). Tumor growth curves of CT26 xenografts after different treatments. b) Representative CD206 and CD11b mIHC images of tumors from the mice treated with siCtrl‐NPs and siSLITRK4‐NPs. Scale bar, 100 µm. Representative Ki67 and CD8 mIHC images of tumors from the mice treated with siCtrl‐NPs and siSLITRK4‐NPs. Scale bar, 50 µm. The comparison of the density of CD8+ki67+cells in iCtrl‐NPs and siSLITRK4‐NPs group. The number of CD206+CD11b cells present relative to the total number of CD11b cells. c) In vivo bioluminescence imaging of the SW620 CRC liver metastasis xenograft model with siCtrl or siSLITRK4‐NPs. d) Quantitative analysis of bioluminescence signals at day 49. Data are presented as the mean ± SD (n = 3 mice in the Ctrl and siCtrl group, and n = 4 mice in the siSLITRK4‐NPs group). e) Representative images of immunofluorescence staining for Ki67 in mouse liver metastases. Scale bar, 50 µm. f) The effect of siSLITRK4‐NPs on the expression of SLITRK4 was evaluated by Western blot. Fold change was computed by comparison with one of the siCtrl‐NPs groups. g) Zoomed‐in bright‐field image of CRC liver metastasis‐derived organoid, KOCO‐164S3, and representative IHC result showed the KOCO‐164S3 organoid showed high expression of SLITRK4. Scale bar, 50 µm. h) Representative images of KOCO‐164S3 tumor xenografts harvested 30 days after different treatments.

To explore the effect of siSLITRK4‐NPs on the immune microenvironment, CT26 tumor‐bearing mouse models were generated and administered with siSLITRK4‐NPs (**Figure** **6**a), then we examined the T cell and macrophage populations and found that siSLITRK4‐NPs decreased the abundance of CD206^+^CD11b^+^ macrophages and increased the abundance of CD8^+^Ki67^+^ macrophages (Figure 6b). We further evaluated siSLITRK4‐NPs in a liver metastasis SW620‐luc xenograft model. First, the establishment of the liver metastasis model was confirmed successfully on day 10 by in vivo bioluminescence imaging. After that, PBS, siCtrl‐NPs, or siSLITRK4‐NPs were administered every other day via tail vein injection. By quantitatively analyzing the bioluminescence signal, we found siSLITRK4‐NPs remarkably reduced CRC liver metastases compared with the control groups (Figure 6c,d). siSLITRK4‐NPs treatment led to obvious decrease in SLITRK4 expression and a reduction in Ki‐67‐positive tumor cells in liver metastases (Figure 6e,f). Thus, targeting SLITRK4 inhibited macrophage polarization and CRC metastasis to the liver. To facilitate the translation of siSLITRK4‐NPs to the clinic, we further evaluated the antitumor efficacy in patient‐derived tumor organoid (PDO)‐derived xenograft (PDOXs) (Figure 6g), as shown in Figure 6h, the tumor volume were remarkably decreased in the siSLITRK4‐NPs group. Taken together, our studies reveal that SLITRK4 acts as a pro‐metastatic factor in liver metastasis formation and inhibition of SLITRK4 possesses a promising clinical translational prospect.

## 3 Discussion

SLITRK family members are a kind of LRR‐containing neuronal transmembrane proteins, and their structural domains are similar to NTRK family members in the extracellular region. Therefore, they are named as NTRK‐like family members. SLITRK family members are known as synaptic organizers. NTRK also plays important roles in the differentiation, growth, and survival of neurons, particularly in the plasticity of synapses in the central and peripheral nervous systems of mammals. Gradually, NTRK overexpression and fusion have been reported in many types of cancer,^[^ ^22^, ^23^ ^]^ leading to tumourigenesis and tumor progression through activation of MAPK and PI3K pathways.^[^ ^24^ ^]^

In recent years, the expression of the SLITRK family has also been investigated in other cells and tissues. For example, SLITRK4 and SLITRK5 are mainly expressed on progenitor cells, immature human hematopoietic stem cells as well as HUVECs.^[^ ^25^ ^]^ Our current study demonstrated for the first time that inhibition of SLITRK4 reduced CRC liver metastasis in vitro and in vivo. In this study, we performed differential gene analysis among paracancerous, primary CRC, and liver metastasis tissues to identify SLITRK4 as a top protumorigenic candidate, which was further confirmed by an additional 21 matched primary CRC and liver metastasis samples. Furthermore, using SLITRK4‐knockout and SLITRK4‐overexpressing CRC cell lines and mouse models, we confirmed that SLITRK4 exerted protumorigenic effects by enhancing tumor growth, invasion, and liver metastasis. Mechanistically, SLITRK4 mediated CRC metastatic growth through several mechanisms,^[^ ^1^ ^]^ regulating PI3K/AKT signaling pathway in cell proliferation and survival,^[^ ^2^ ^]^ promoting tumor angiogenesis by upregulating VEGFA,^[^ ^3^ ^]^ regulating extracellular matrix and cell‐matrix adhesion molecules, and^[^ ^4^ ^]^ enhancing TAM infiltration and polarization, releasing tumor growth‐promoting factors and cytokines and further inducing immune tolerance.

Once cancer cells disseminated from the colon are lodged in the liver, their ability to secrete cytokines and molecules is enhanced to induce angiogenesis and tumor growth.^[^ ^26^ ^]^ However, the potential mechanism remains unclear. In our study, the knockdown and overexpression of SLITRK4 in CRC cells suggested that SLITRK4 could regulate the expression of ECM molecules and cytokines via the PI3K/AKT and NF‐κB pathway(Figure 3). Sequence analysis suggested that the extracellular two leucine‐rich repeat (LRR) domains of SLITRKs, and a conserved region in the intracellular carboxyl terminus have a high degree of consensus with the last 16 amino acids of NTRK, encoded tropomyosin‐related kinase (TRK).^[^ ^6^ ^]^ NTRK aberrations, such as overexpression or gene fusion, have been implicated in the pathogenesis of many cancer types.^[^ ^27^ ^]^ The activation of NTRK leads to several downstream signaling pathways, including PI3K/Akt, MEK/ERK, and NFκB.^[^ ^28^, ^29^ ^]^ Therefore, SLITRK4 overexpression showed similarity with the NTRK‐like pathway.

ECM, a highly active component of the TME, influences tumor growth and migration. Emerging evidence shows that COMP has a positive relationship with poor survival in a variety of cancers, including breast cancer, CRC, and hepatocellular carcinoma.^[^ ^30^, ^31^, ^32^ ^]^ COMP governs cytoskeletal remodeling and promotes CRC cell metastasis via interacting with the actin‐binding protein transgelin (TAGLN) in the EMT process.^[^ ^31^ ^]^ Consistently, COMP overexpression existed in CRC patients and was correlated with poor progression of CRC, and importantly, the expression of COMP and the EMT process were significantly regulated by SLITRK4.

Macrophage recruitment into the tumor stroma is crucial for tumor progression and metastasis via enhancement of cell proliferation, matrix remodeling, angiogenesis, and immune escape.^[^ ^33^ ^]^ Tumor immune infiltration as explored by TIMER suggested that SLITRK4 is correlated with macrophage abundance. The cytokines CCL2, CCL8 (IL‐8), and CSF1 produced by CRC cells were remarkably induced by SLITRK4 via PI3K/AKT/NF‐κB (Figure 3; Figure S3a, Supporting Information). Accumulated evidence suggests that TAMs infiltration is an independent poor prognostic factor in multiple types of cancer.^[^ ^34^ ^]^ W. Tu et al. found that TAM infiltration was enhanced in CRC with liver metastasis compared with primary tumors, which was correlated with a worse prognosis.^[^ ^35^ ^]^ CCL2 contributes to CRC progression by promoting monocyte chemoattraction and TAMs infiltration.^[^ ^36^ ^]^ In addition, metastasis‐associated macrophages can also be recruited by tumor cell‐derived CCL2 and the CCL2‐CCR2 pathway. Moreover, CCL8 is another chemokine ligand of CCR2, which also promotes TAMs infiltration. Activation of CCR2 in macrophages is essential for their polarization toward a pro‐tumor phenotype.^[^ ^37^ ^]^ CSF‐1 is a major survival factor for TAMs and triggers TAMs polarization by interacting with the CSF1 receptor.^[^ ^38^ ^]^ Blocking the CCR2 receptor and CSF1 receptor, respectively, could significantly reduce SLITRK4 overexpression‐induced TAMs infiltration and polarization (Figure 4c). Taken together, overexpression of SLITRK4 could upregulate CRC cell‐derived CCL2, CCL8, and CSF1 production to promote both TAMs recruitment and polarization.

Using the coculture system, we further confirmed that SLITRK4 expression in cancer cells is related to TAMs infiltration and polarization (Figure 4). Transwell assays suggested knockdown of SLITRK4 led to a decreasing infiltration ability of macrophages (Figure 4b). By detecting macrophage polarization‐related chemokines, we identified IL10 as a potential downstream target in THP1 cells treated with SW480‐ovSLITRK4 cells and SW620‐shSLITRK4 cells (Figure 4d). IL‐10 is a typical anti‐inflammatory cytokine that acts as a negative mediator of immune responses.^[^ ^39^ ^]^ In addition, flow cytometry also pinpointed that overexpressing SLITRK4 promoted PBMCs cells with a significant bias toward CD206^high^CD86^low^ (Figure 4e). scRNA‐seq confirmed that STLITRK4 promoted immunosuppression status (Figure S4, Supporting Information; Figure 4 g,h). In addition, the downregulation of SLITRK4 could significantly activate an antitumorous CD8^+^ T‐cell response (Figure 4h). Combined with the present results, we, therefore, hypothesized that high expression of SLITRK4 is correlated with monocyte‐derived macrophage infiltration and polarization, which may further contribute to the immune escape of tumor cells. Therefore, targeting SLITRK4 may trigger effective remodeling of the TME to an anti‐tumor state. A more detailed catalog of the specific macrophage and T cell regulation that require SLITRK4 for optimal signal transduction will require further investigation.

As the majority of patients with CRC liver metastasis have no access to curative resection, conversion therapy is the main therapeutic approach to convert unresectable lesions into resectable nidus.^[^ ^40^ ^]^ However, there is a lack of effective drugs for conversion therapy. The clinical benefit of targeted drugs, such as cetuximab, is limited due to side effects and drug resistance. Moreover, the efficacy of immune checkpoint inhibitors is also undesirable because only 5% of CRC patients with metastases have high microsatellite instability (MSI‐H)/mismatch repair defect (dMMR).^[^ ^41^ ^]^ Recent advances in siRNA nanomedicines with good potency and safety have demonstrated the potential of developing an NP platform for the delivery of siRNA targeting SLITRK4. Therefore, targeting SLITRK4 might be a promising new strategy for conversion therapy.

As an emerging generation of siRNA delivery vehicles, lipid‐polymer hybrid nanoparticles integrate the advantages of lipid‐like materials and polymers and exhibit several unique features. Compared with siRNA lipoplexes or polymer/siRNA complexes, the solid polymer/cationic lipid core of our hybrid NPs provides a stable structure for siRNA encapsulation, which can better protect siRNA from degradation, while the PEG shell prolongs the circulation of the hybrid NPs in the bloodstream, which may favor their accumulation at the tumor sites due to the enhanced permeability and retention effect. The as‐prepared siSLITRK4‐NPs showed a uniform spherical structure (Figure 5b,c). Our results demonstrated that siSLITRK4‐NPs could mediate successful siSLITRK4 delivery in colon cancer cells for growth inhibition (Figure 5d,e). Moreover, the therapeutic effect of siSLITRK4‐NPs was further confirmed in xenograft, liver metastasis models, and PDOX models (Figure 5g–k and Figure 6).

In summary, we found a newly pro‐oncogenic role of SLITRK4 in exacerbating CRC tumorigenesis and metastasis. Inhibition of SLITRK4 could suppress CRC liver metastasis by modulating cell‐matrix adhesion, reducing angiogenesis and TAM infiltration in the TME. Furthermore, our study pinpointed the potential of siSLITRK4‐NPs for CRC treatment.

## 4 Experimental Section

### Cell culture

The human CRC colorectal cell lines (LoVo, Caco‐2, SW620, SW480, and HCT116), mouse CT26, and human THP‐1 cells were obtained from Shanghai Cell Bank, Institute of Biochemistry and Cell Biology, Chinese Academy of Sciences (Shanghai, China). THP‐1 cells were cultured in RPMI 1640 medium, 10% FBS, and 1% penicillin/streptomycin. Other cells were grown in DMEM supplemented with 10% fetal bovine serum and 1% penicillin/streptomycin. All cells were cultured at 37 °C in a humidified atmosphere supplemented with 5% CO_2_.

### Colorectal Cancer Patient Specimen

The study was approved by the Biomedical Research Ethics Committee of Peking University First Hospital, and the authorization number was 2018‐15. All human samples, including 4 adjacent normal tissues (> 5 cm away from the tumor), 3 primary CRC, and 6 liver metastasis samples from 8 patients, were collected from patients with written informed consent. Clinical parameters including sex, age, stage, infiltration, location, and differentiation were shown in Table S1 (Supporting Information). All patients had not received local or systemic treatments before the operation. Fresh tumors and normal tissues were frozen in liquid nitrogen and stored at −80 °C for further RNA‐Seq analysis.

### Cell Viability Assay

SW620, SW480, and HCT116 cells were inoculated in 96‐well culture plates at a 4 × 10^3^/well concentration and cultured overnight. Incubation was continued for 24 h after the addition of the compound. Finally, CCK8 and MTT were added and incubated for 4 h. The absorbance values of the CCK8 viability assay were recorded at 450 nm, and the MTT assay was recorded at 490 nm.

### Colony Formation Assay

First, the 96‐well plate was coated with 20 µg mL^−1^ fibronectin (Sigma, F0895) at 4 °C. After 12 h of coating, fibronectin was removed, and the cells were blocked with 1% bovine serum albumin for 1 h at 37 °C and washed 3 times with PBS. Cells (2 × 10^5^ mL^−1^) were seeded on a preprepared 96‐well plate in a warm bath serum‐free medium. After 4 h of incubation, the cells were washed 3 times with PBS to remove the inadvertent cells, fixed in 4% paraformaldehyde and stained with Hoechst. Cells from 5 random fields (at × 10 magnification) were counted under a microscope.

### Cell Adhesion Assay

First, the 96‐well plate was coated with 20 µg mL^−1^ fibronectin (Sigma, F0895) at 4 °C. After 12 h of coating, fibronectin was removed, and the cells were blocked with 1% bovine serum albumin for 1 h at 37 °C and washed 3 times with PBS. Cells (2 × 10^5^ mL^−1^) were seeded on a pre‐prepared 96‐well plate in warm bath serum‐free medium. After 4 h of incubation, the cells were washed 3 times with PBS to remove the inadvertent cells, fixed in 4% paraformaldehyde, and stained with Hoechst. Cells from 5 random fields (at × 10 magnification) were counted under a microscope.

### Cell Invasion and Migration Assay

Cell invasion and migration were evaluated by 24‐well Transwells (8 µm pore size, Corning, USA) according to the manufacturer's instructions. For the cell invasion assay, the upper chambers were precoated with Matrigel, and the upper chambers in the cell migration assays were uncoated with Matrigel. In total, a cell suspension containing 1 × 105 cells in 200 µL DMEM without FBS was placed in the upper chamber, while 600 µL DMEM containing 10% FBS was added to the lower chamber. After incubation for 48 h, the cells were fixed in 4% paraformaldehyde and stained with 0.5% crystal violet. An inverted microscope was used to capture the image of invasion or migrated cells stained with 0.5% crystal violet. Finally, the number of cells in four random microscopic fields was counted and averaged. For the macrophage migration assays, a total of 5 × 10^5^ tumor cells were plated in the 6‐well culture plates containing CCR2 inhibitor RS102895 (20 µM, Selleck, USA), GDF15 antibody inhibitor Ponsegromab (10µg mL^−1^, Selleck, USA) and CSF‐1 neutralizing antibody Lacnotuzumab (10µg mL^−1^, Selleck, USA) or DMSO for 24h. After replacing it with a fresh medium, the culture was continued for 24 h, and then the supernatant was collected. THP‐1 cells were cultured in 6‐well culture plates (5 × 10^5^/well) containing PMA (100ng mL^−1^, Sigma, USA) for 48 h. The cell culture medium was replaced with that collected above to continue culturing for 48 h. Macrophages were collected for migration assay in the same steps as described above.

### Tube Formation Assays

A Matrigel‐coated ibidi angiogenesis slide (ibidi GmbH) was applied to investigate the tube formation of HUVECs. Conditioned medium derived from transfected SW480 and SW620 cells was collected after 48 h and mixed as angiogenesis assay medium (10% endothelial cell medium, 90% conditioned medium). A total of 1 × 10^4^ primary HUVECs (P2–P6) were placed in 50 µL of angiogenesis assay medium. At 6 h after seeding, images were obtained by light microscopy. Wimasis fully automated analysis was used to measure tubular length and the number of loops.

### Preparation of Primary Human PBMC‐Derived Macrophages

Primary human PBMCs were separated and cultured as previously described. 20 mL of elbow vein blood from 6 healthy volunteers with informed consent, approved by the Biomedical Research Ethics Committee of Peking University First Hospital (authorization number was 2024–328). Briefly, PBMCs were isolated from peripheral blood using a human lymphocyte isolation solution, and monocytes were separated using CD14 magnetic beads. The concentration of monocytes was adjusted to 1 × 106/ml. Then, induction of monocyte macrophage maturation by adding M‐CSF (25ng mL^−1^) to RPMI 1640 media. After 5 days, the cells were activated with cellular supernatant.

### Flow Cytometry Analysis

For all experiments, macrophage cells were first stained with Human TruStain FcX (Fc Receptor Blocking Solution, biolegend) in 1% BSA for 5–10 min at room temperature followed by surface antibody staining. The following antibodies were used, FITC anti‐human CD11b, PE/Cyanine7 anti‐human CD86, PE anti‐human CD206 (Biolegend).

### Western Blotting

Total protein was extracted from SW480, SW620, and their transfection cell lines, and examined by Western blot following the previously published protocols.^[^ ^42^ ^]^ In brief, cells or tumor tissue were lysed on ice with RIPA buffer and centrifuged at 4 °C. Equivalent amounts of protein (50 µg) were separated by 10% SDS‒PAGE and transferred to PVDF membranes (Millipore, Bedford, MA, USA). The membranes were blocked with 5% milk in TBST and then incubated with primary antibodies overnight at 4 °C. After washing with PBS, the membranes were incubated with HRP‐conjugated goat‐anti‐mouse or goat‐anti‐rabbit secondary antibodies. The immunoreactive bands were visualized with BeyoECL Plus (Beyotime, P0018S). The various cytokine levels were determined by Luminex assays (R&D Systems).

### qRT‒PCR

Total RNA was extracted from CRC colorectal cell lines by TRIzol reagent (Invitrogen, MA, USA) and reverse‐transcribed into cDNA via a Color Reverse Transcription Kit (EZBioscience, MN, USA). Then, the qRT‒PCR assay was conducted on an Applied Biosystems 7500 Real‐Time PCR System (Applied Biosystems, CA, USA). Relative expression of GAPDH was calculated as the internal control by the 2^−ΔΔCt^ method.

### ELISA

IL‐8 and VEGFA were analyzed using ELISA kits (Hangzhou Lianke Biology Technology Co., Ltd.) according to the manufacturer's instructions.

### Luminex Cytokine Assays

The mouse Cytokine/Chemokine Magnetic Bead Panel kit was purchased from Bio‐RAD (cat. no. M60009RDPD). Assays in the 96‐well format were conducted on filter plates based on the manufacturer's recommendations. In total, 200 µL of wash buffer was added to each well of a 96‐well plate. The plate was sealed and mixed on a plate shaker for 10 min at room temperature (20–25 °C). Then, the 96‐well plate was placed on a Luminex Magnetic Plate Separator and incubated for 1 min. The wash buffer was removed. Then, 50 µL of each standard, control, and sample was added to each well, 20 µL of tissue lysis fluid was added to each standard and control well, and 25 µL of assay buffer was added to each sample well. The working bead mix was vortexed immediately before use. Next, 50 µL of the mixed beads was added to each well. The plate was then sealed, wrapped with aluminum foil, and incubated with agitation on a plate shaker for 1 h at room temperature. After incubation, the liquid was removed from the plate. The plate was washed and removed twice with 200 µL of wash buffer each time. After the second wash, 50 µL of detection antibodies were added to each well. The plate was then sealed, wrapped in aluminum foil, and incubated with agitation on a plate shaker for 1 h at room temperature. Next, 50 µL of streptavidin‐phycoerythrin was added to each well containing 50 µL of detection antibodies. The plate was shaken for an additional 30 min at room temperature. The liquid was removed from the plate. The plate was washed and removed twice with 200 µL of wash buffer each time. The bottom of the plate was dried by tapping on a paper towel. Then, 150 µL of sheath fluid (cat. no. 40–50000, Luminex) was added to each well. The beads were resuspended on a plate shaker for 5 min and read on a Luminex 200 instrument (Luminex, CA). The instrument was set to collect at least 50 beads per analyte. The raw data were analyzed by Milliplex Analyst software and measured as the mean fluorescence intensity (MFI).

### Immunohistochemical Staining and Polychromatic Immunofluorescence Staining

The details of the immunohistochemical staining procedures are described in the previous study. Briefly, sections were blocked with 3% normal goat serum and incubated with primary antibodies overnight at 4 °C. The primary antibodies used for immunohistochemical staining were Ki67 and CD31 (Abcam, Cambridge, UK). Sections were visualized with suitable secondary antibodies followed by avidin‐biotin‐peroxidase complex staining. Polychromatic immunofluorescence staining was conducted by utilizing the four color multiple fluorescent immunohistochemical staining kit (abs50012, Absin, Shanghai, China) referring to the tyramide signal amplification (TSA) technique according to the manufacturer's manual.

### In Vivo Mouse Assays

Total BALB/c‐nude mice (6–8 weeks old, male) and BALB/c mice (6–8 weeks old, male) were obtained from Beijing Huafukang Biotechnology Co., Ltd. and housed in the animal care facility. The animal use protocol was approved by the Institutional Animal Care and Use Committee of Peking University Health Science Center. The in vivo xenograft and metastasis assays were carried out based on previous protocols.^[^ ^43^ ^]^ SW480, SW620, and CT26 cells transfected with shNC, shSLITRK4, ovNC, or ovSLITRK4 were inoculated into the left flank of mice or intrasplenic injection of mice to establish in vivo tumor growth or liver metastasis models, respectively. Liposome‐clodronate/PBS (1 mg/mouse, twice a week) was administered by i.v. injection. Noninvasive real‐time imaging of tumors in mice was performed by an IVIS Spectrum fluorescence imager.

### Preparation of Lipid‐Polymer Hybrid Nanoparticles for siSLITRK4 Delivery

Poly(ethylene glycol) methyl ether‐block‐poly(L‐lactide‐co‐glycolide) (mPEG_5K_‐PLGA_15K_ (LA/GA = 50/50)) was purchased from Xi'an Ruixi Biological Technology Co., Ltd. The cationic lipid‐like compound G0‐C14 was synthesized through a ring opening reaction of 1,2 epoxytetradecane with PAMAM‐G0 according to previously described methods. The copolymer mPEG5K‐PLGA15K and cationic lipid‐like compound G0‐C14 were used to encapsulate siSLITRK4 into lipid‐polymeric hybrid nanoparticles by a one‐step nanoprecipitation method. Briefly, mPEG_5K_‐PLGA_15K_ and G0‐C14 were first dissolved separately in dimethylformamide at concentrations of 5.0 mg mL‐1. Then, 15.0 µL of siRNA (1.5 nmol) (siSLITRK4, siCtrl, or Cy5‐labeled siSLITRK4) aqueous solution was mixed with 400 µL of mPEG_5K_‐PLGA_15K_ (2.0 mg) and 100 µL of G0‐C14 (0.5 mg) solutions. The above mixture was added dropwise to 10 ml of DNase/RNase‐Free HyPure water (Invitrogen) under vigorous stirring (1000 rpm) and then kept for another 10 min. After that, the formed nanoparticles were transferred to ultrafiltration tubes (molecular weight cutoff = 100 kDa, Millipore Co., Billerica, MA, USA) and centrifuged to remove the organic solvent and unencapsulated siRNA. After washing twice with HyPure water, the siRNA‐encapsulated NPs were collected and dispersed in pH 7.4 PBS buffer for further use or stored at −80 °C.

### Physicochemical Characterization of siRNA‐Encapsulated NPs

The size and size distribution of the above siRNA‐encapsulated NPs were determined by DLS technology using a Malvern Zetasizer Nano ZS instrument at 25 °C. The zeta potentials were also measured. The morphological structure was visualized by a Talos F200S G2 transmission electron microscope (TEM, Thermo Scientific). To determine the siRNA encapsulation efficiency (EE%), 5 µL of Cy5‐labeled siSLITRK4‐NPs solution was added to 100 µL of dimethyl sulfoxide, and the fluorescence intensity was measured by a BioTek Synergy HT multimode microplate reader (Winooski, VT, USA) (λex/λem = 633/670 nm). Free Cy5‐labeled siSLITRK4 was used as a standard. The EE% of Cy5‐labeled siSLITRK4NPs was calculated to be 48%.

### Cellular Uptake Study

SW620 cells were seeded onto 6‐well plates at a density of 1.5 × 10^5^ per well and cultured for 24 h. Cy5‐labeled siSLITRK4‐NPs or free Cy5‐labeled siSLITRK4 were then added at a siRNA concentration of 20 nM. After further incubation for 0.5, 2, or 4 h, the cells were harvested for flow cytometry analysis on FACS caliber flow cytometer (FACSCalibur, BD Biosciences, San Jose, CA, USA).

### Tumor Inhibition Study in a Subcutaneous Xenograft Model

The HCT116 tumor bearing mouse model was established by subcutaneous injection of HCT116 cells into the left flank of BALB/c‐nude mice (6–8 weeks old, male). CT26 cells tumor bearing model was built by subcutaneous injection of CT26 cells into the left flank of BALB/c mice (6–8 weeks old, male).When the tumor grew to a defined volume, the mice were randomly divided into three groups and then administered with PBS, siCtrl‐NPs, or siSLITRK4‐NPs via tail vein injection at a fixed siRNA dose of 1.0 nmol per mouse on days 1, 3, 6, and 7. The tumor volume and the body weight were recorded every 2 days during the treatment. At the endpoint, the mice were sacrificed, and the tumors were collected. In addition, the SW620 tumor bearing mouse model was established as a similar protocol for dose‐dependent anti‐tumor evaluation of siSLITRK4‐NPs. Mice were randomly divided into four groups and were then administered with PBS or siSLITRK4‐NPs of different doses (0.5 nmol per mouse, 1.0 nmol and 2.0 nmol per mouse) on days 1, 3, 6, and 7. The tumor volume and the body weight were recorded every 2 days during the treatment. At the endpoint, the mice were sacrificed, and the tumors were harvested. Humanized NOD‐Prkdc^em1Idmo^‐Il2rg^em2Idmo^ (Hu‐NPI) mice (21–22 weeks old, male) were purchased from BEIJING IDMO Co., Ltd. CRC liver metastasis‐derived organoid, KOCO‐164S3, were provided from K2 ONCOLOGY Organoids Platform. KOCO‐164S3 harvested using cell recovery solution, which could completely dissolve BME (Amsbio) while keeping organoids intact. KOCO‐164S3 organoids were resuspended with 5 mL of DPBS after centrifugation at 400 ×g for 5 min. 50 µL of the organoid was then taken from 5 mL and proceeded to digestion into single cells with TrypLE for cell counting.Then, the organoids were resuspended in 50% Matrigel. 100 µL of the mixture was injected into the subcutaneous of the right forelimb of Hu‐NPI mice with a 1 mL sterile syringe. The rate of tumor formation differed from organoids and the tumor size was routinely monitored. When the tumor grew to 150–200 mm^3^, siSLITRK4‐NPs treatment started. Mice were euthanized after a maximum of 36 days of treatment.

### Tumor Inhibition Study in a Liver Metastasis Xenograft Model

The liver metastasis xenograft model was established by intrasplenic injection of SW620‐luc cells in BALB/c‐nude mice. After confirmed the successful growth of the liver metastasis tumor on day 10 by in vivo bioluminescence imaging, the mice were randomly divided into three groups and then administered with PBS, siCtrl‐NPs, or siSLITRK4‐NPs via tail vein injection at a fixed siRNA dose of 1.0 nmol per mouse on days 1, 3, 6, and 7. At days 44 and 47, the mice were imaged under an IVIS Spectrum fluorescence imager. At the endpoint, the mice were sacrificed, and the liver tissues were collected for hematoxylin‐eosin (HE) staining and immunofluorescence analysis of SLITRK4 and Ki‐67.

### Single‐Cell RNA‐Seq Analysis

Surgically resected tumor tissue obtained from 3 mice was digested into single‐cell suspensions, and then cell capture and cDNA synthesis were performed on a Chromium single‐cell controller (10x Genomics). After passing all quality controls, transcriptome sequencing was carried out on an Illumina NovaSeq 6000 sequencer. Cell Ranger software was used based on the 10x Genomics website for data alignment, filtering, barcode counting, and UMI counting to generate a feature‐barcode matrix and determine clusters. Then, PCA was performed for dimensionality reduction, and the principal components were used to generate clusters based on the K‐means algorithm and graph‐based algorithm. R package Seurat 3.0. Cells were also used for other clustering. singleR was performed for cell type annotation based on unbiased cell type recognition by utilizing reference transcriptomic datasets of pure cell types to deduce the cell origin of each single cell. Blueprint Encode or HPCA was used for humans.

### Statistics

All data were expressed as the means ± standard deviations (S.D.) from at least three independent experiments. Statistical analyses were performed by Graphpad Prism 10.1.2 using the two‐tailed Student's *t*‐test to detect differences between the groups. P values less than 0.05 were considered statistically significant.

## Conflict of Interest

The authors declare no conflict of interest.

## Author Contributions

X.S., J.Z., B.D., and Q.X. contributed equally to this work. X.J.S., J.L.Z., and B.Q.D. performed the main cell and mouse experiments. B.Q.D. undertook TCGA analysis and western blotting validation assays. Q.Q.X. and Y.L.G. prepared nanoparticles for SLITRK4 siRNA delivery and prepared the manuscript. J.L.Z. and X.W. provided clinical samples. J.L.Z. and X.H. performed the statistical analysis of the manuscript. X.C.P., Y.M.C., and Z.M.L. provided funding for the study. X.C.P., Y.M.C., and Z.M.L. contributed to in the design, coordination and manuscript editing. All authors have read and approved the final manuscript.

## Supporting information



Supporting Information

## Data Availability

The data that support the findings of this study are available from the corresponding author upon reasonable request.,
