# Supplementary material for: Targeting SLITRK4 Restrains Proliferation and Liver Metastasis in Colorectal Cancer via Regulating PI3K/AKT/NFκB Pathway and Tumor‐Associated Macrophage
Source: Adv Sci (Weinh). 2024 Nov 5;12(1):2400367. doi: 10.1002/advs.202400367 (PMC11714161; doi:10.1002/advs.202400367)
Supplement: Supplementary file 1 — Supporting Information [file ADVS-12-2400367-s001.docx]

Supporting Information

**Targeting SLITRK4 Restrains Proliferation and Liver Metastasis in Colorectal Cancer Via Regulating PI3K/AKT/NFκB Pathway and Tumor-associated Macrophage**

Xiaojiao Sun^1#^, Junling Zhang^2#^, Bingqi Dong^2#^, Qingqing Xiong^3#^, Xin Wang^2^, Yanlun Gu^1,4,5^, Zhiqi Wang^1^, Huiyu Liu^1^,Jixin Zhang^6^, Xu He^4,5^, Hongjin Liu^2^, Yi Zhong^1^, Chuxiao Yi^1^, Xiaowei Chi^1^, Zhenming Liu^1*^, Xiaocong Pang^1,4,5*^,Yimin Cui^1,4,5*^

^1^State Key Laboratory of Natural and Biomimetic Drugs, School of Pharmaceutical Sciences, Peking University, Beijing, China

^2^Department of General Surgery, Peking University First Hospital, Xishiku Street, Xicheng District, 100034 Beijing, China

^3^Department of Hepatobiliary Cancer, Liver Cancer Center, Tianjin Medical University Cancer Institute, 300060 Tianjin, China

^4^Department of Pharmacy, Peking University First Hospital, Xishiku Street, Xicheng District, 100034 Beijing, China

^5^Institute of Clinical Pharmacology, Peking University, Xueyuan Road 38, Haidian District, 100191 Beijing, China

^6^Department of Pathology, Peking University First Hospital, Xishiku Street, Xicheng District, 100034 Beijing, China

**Correspondence**

Address correspondence to: Peking University, Xueyuan Road 38, Haidian District, 100191 Beijing, China; E-mail: zmliu@bjmu.edu.cn (ZML); pangxiaocong1227@163.com(XCP); cui.pharm@pkufh. com (YMC).

^#^These authors contributed equally to this work.

**This file includes:**

Supplementary Figure S1-S5 and Figure Legends

Supplementary Table S1


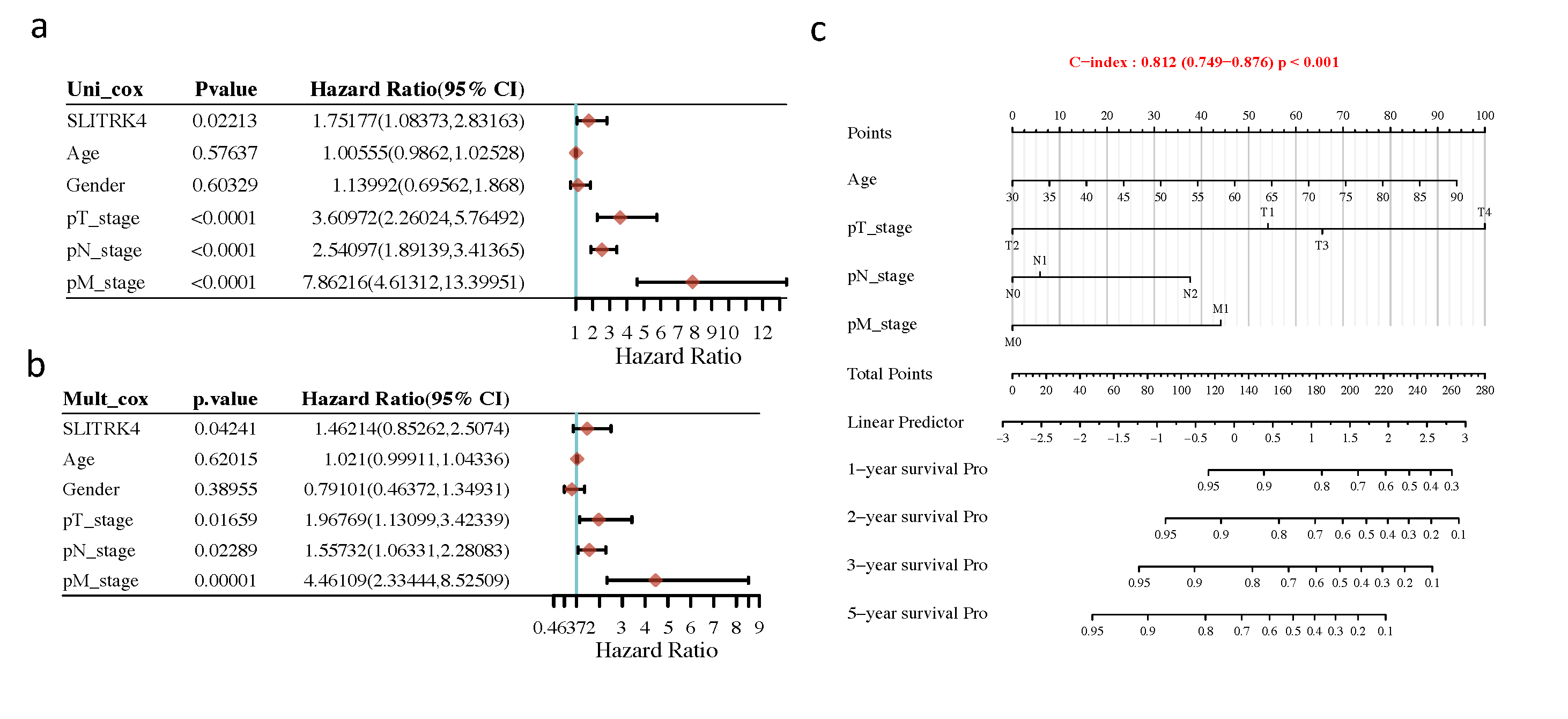


**Figure S1**. SLITRK4 is an independent indicator of patient overall survival. (a, b) The pvalue, risk coefficient (HR) and confidence interval are analyzed by univariate and multivariate Cox regression. (c) Nomogram predicts the 1-year，2-year and 3-year overall survival of CRC cancer patients.


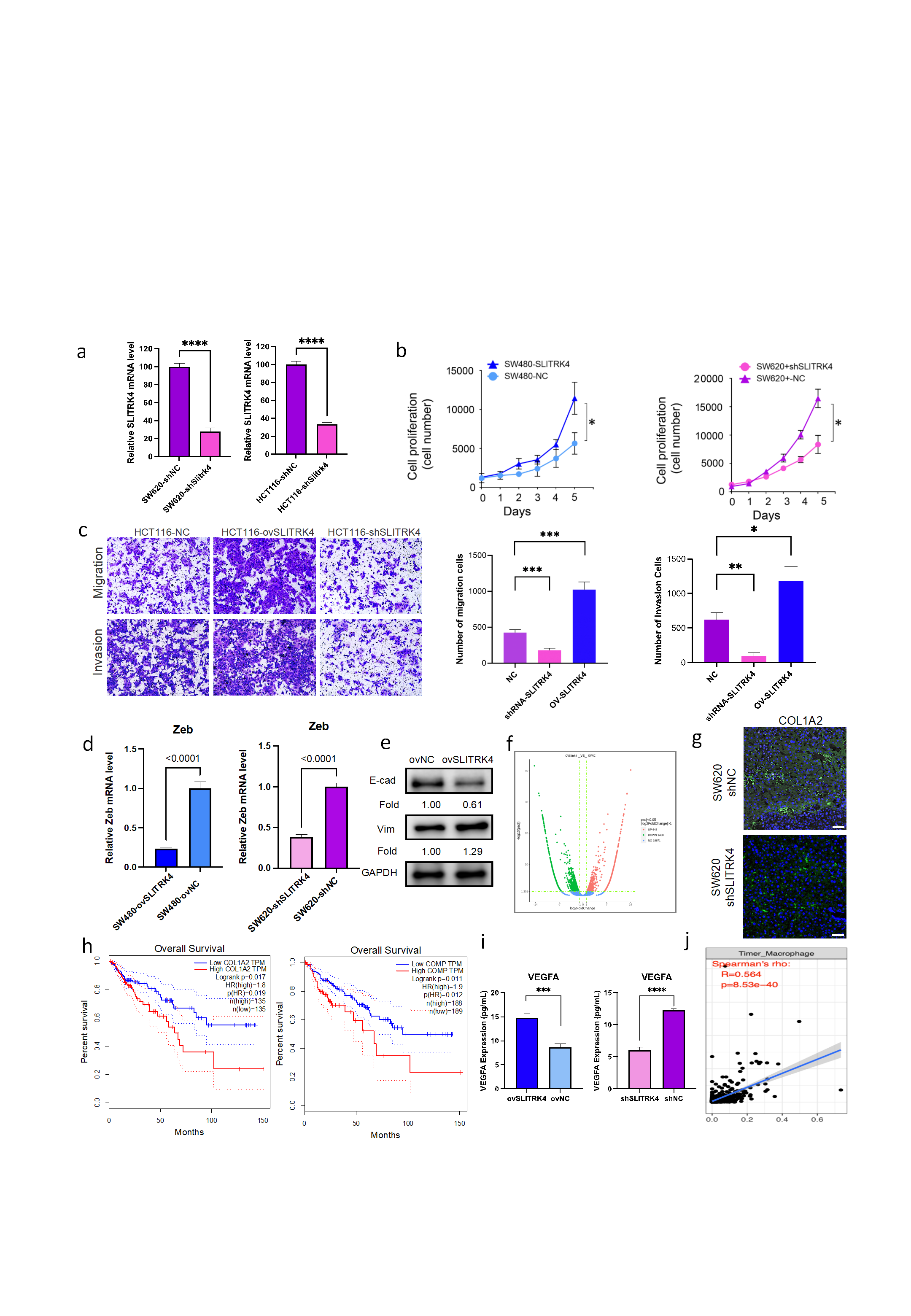


**Figure S2.** SLITRK4 is involved in CRC progression. (a) The proliferation of SW480 and SW620 CRC cells was evaluated by CCK-8 assay. (b) Overexpression of SLITRK4 promoted CT26 colorectal cancer liver tumor progression. (c) The effect of SLITRK4 on HCT116 cell invasion and migration was detected by transwell assay. (Magnification ×100) (d) SLITRK4 significantly regulated the transcription factor Zeb-mediated EMT process. (e) The effect of SLITRK4 on the expression of EMT-related proteins was evaluated by Western blot. (f) Class comparison analysis for DEGs was conducted with the EdgeR Bioconductor package (R version 3.5.1), and DEGs with p < 0.05 and |log2FC| >1.5 were selected for further analysis. (g) The PI3K inhibitor LY294002 inhibited the SLITRK4-mediated proliferation promotion effect. (h) Representative images of of COL1A2 expression liver metastatic tissue from SW620 cells with and without SLITRK4 knockdown. Scale bar, 50 μm. (i) High expression of COL1A and COMP was associated with worse overall survival of CRC patients. (j) SLITRK4 expression was positively correlated with infiltrating levels of macrophages in colon adenocarcinoma (COAD) via TIMER methods. All histogram data are presented as the mean ± SD. n = 3 biological replicates. *P < 0.05; **P < 0.01; ***P < 0.001; ****P < 0.0001.


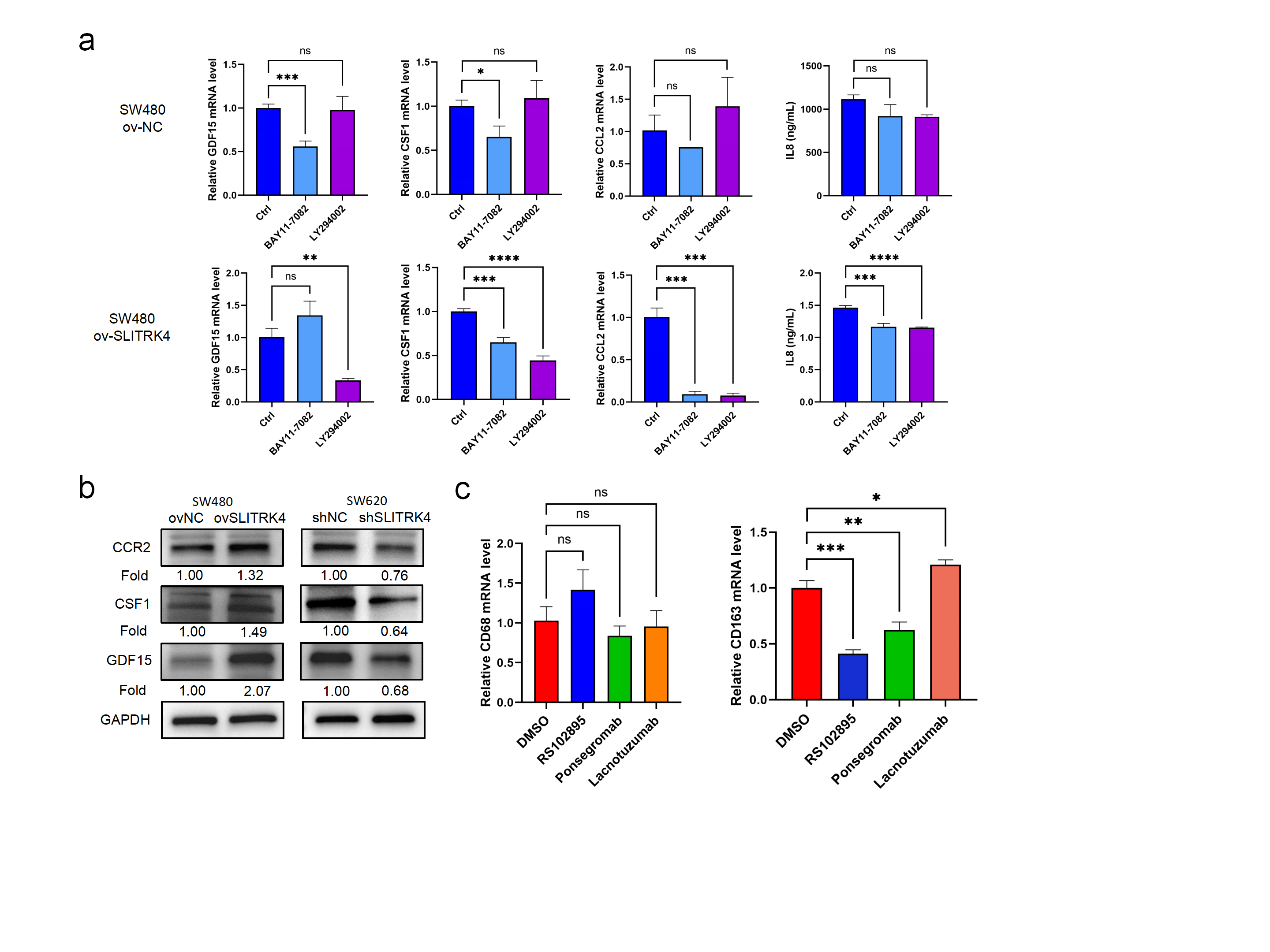


**Figure S3**. SLITRK4 promotes the secretion of TAMs-related cytokines by mediating the PI3K/AKT/NFκB pathway. (a) The effect of PI3K inhibitor LY294002 and NF-κB inhibitor BAY11-7082 on SLITRK4 overexpression induced the secretion of cytokines (CSF1, CCR2, GDF15 and IL8). (b) The effect of SLITRK4 on the expression of TAMs-related cytokines was evaluated by Western blot. Fold change was computed by comparation with ovNC or shNC group. (c) The effect of RS102895, Ponsegromab, and Lacnotuzumab on macrophage associated markers CD68 and CD163. ns, no significance; *P < 0.05; **P < 0.01; ***P < 0.001; ****P < 0.0001.


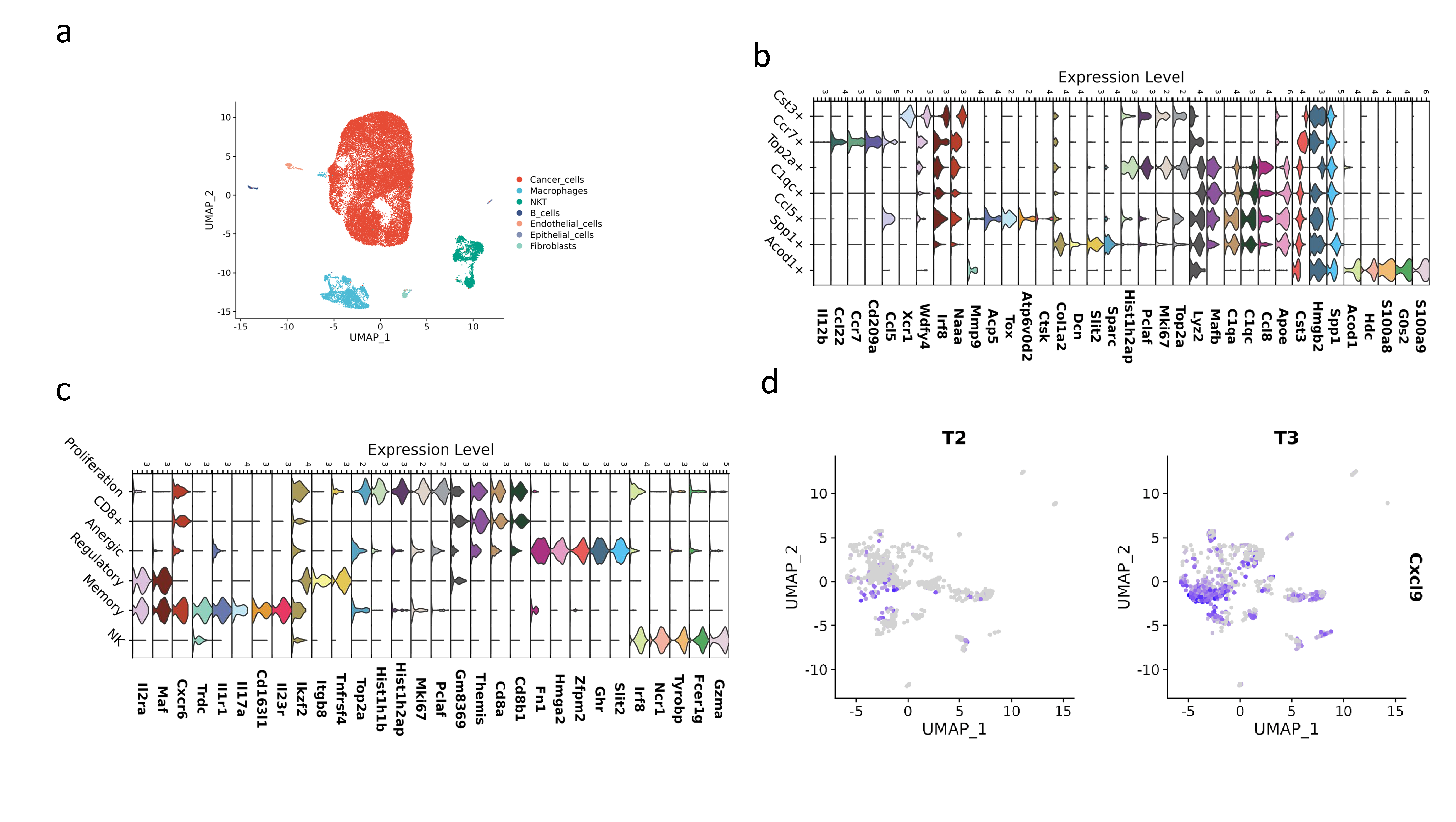


**Figure S4.** Characterizing the single-cell landscape in CT26 colorectal cancer liver tumors. (a) Uniform manifold approximation and projection (UMAP) representation of dimensionally reduced data color-coded by assigned cell type. (b, c) Heatmap describing marker gene expression levels and the percentage of cells expressing the marker. (d) The effect of SLITRK4 on the expression of CXCL9 in TAMs, the STLITRK4 overexpression group (T2), and the STLITRK4 knockdown group (T3).


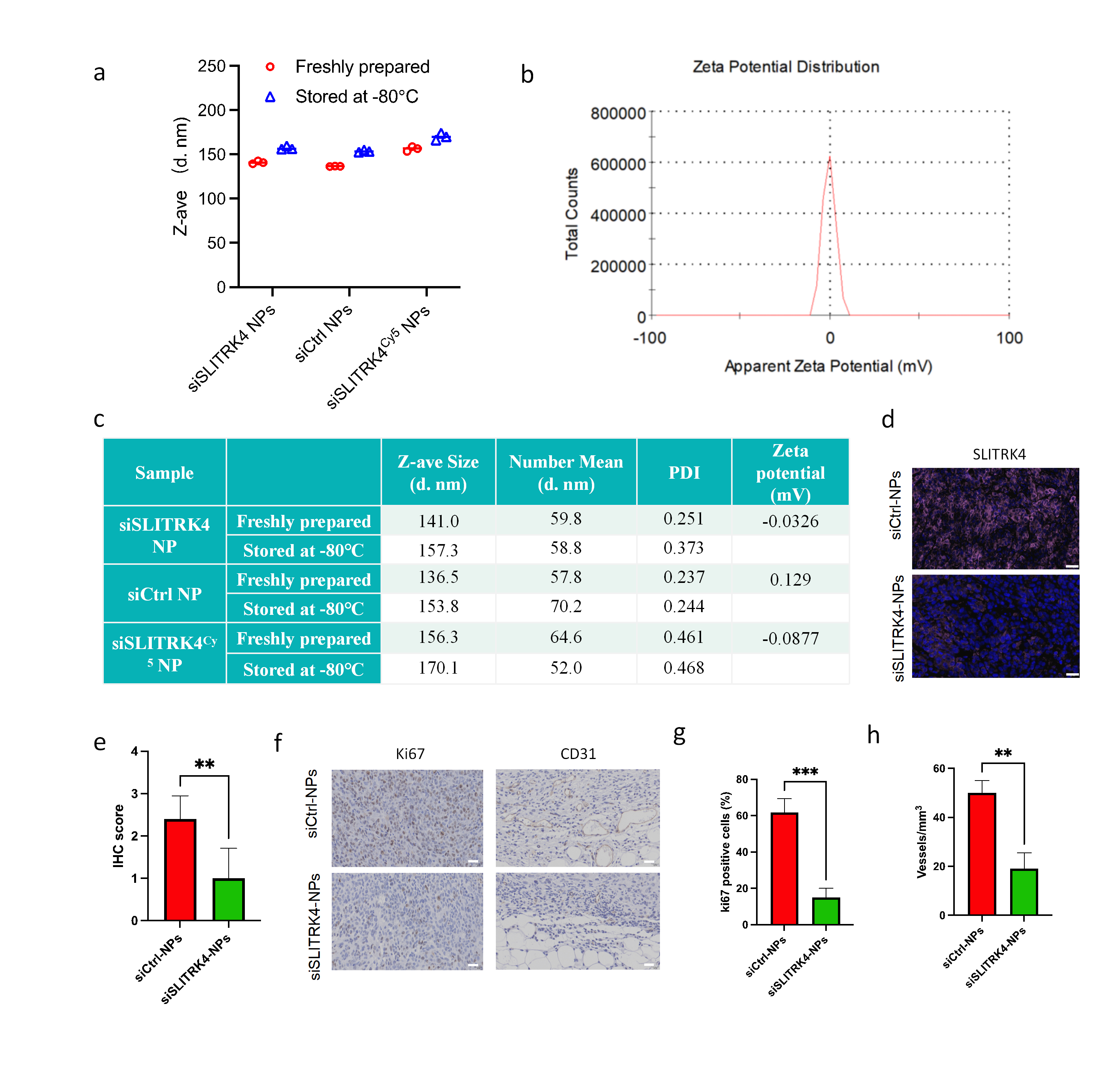


**Figure S5.** Development of siRNA-encapsulated NP for CRC therapy. (a, b, c) The effect of fresh preparation and storage at -80°C on the zeta potential (n=3). (d,e) Representative images of immunofluorescence staining and IHC score analysis for SLITRK4 in mouse liver metastases. Scale bar, 50 μm. (f) Representative Ki67 and CD31 IHC images of tumor from the mice treated with siCtrl-NPs and siSLITRK4-NPs.(g, h) Statistical analysis of Ki67 and CD31. All histogram data are presented as the mean ± SD. n = 3 biological replicates. *P < 0.05; **P < 0.01; ***P < 0.001; ****P < 0.0001.

Table S1. Patient information for RNA-Seq analysis.

| Patient ID | sex | age | Location | Differentiation | Infiltration |
| --- | --- | --- | --- | --- | --- |
| Patient 1 | Male | 55 | Sigmoid colon | moderate | T3N1M0 |
| Patient 2 | Male | 47 | Sigmoid colon | moderate | T2N1M0 |
| Patient 3 | Male | 63 | Sigmoid colon , liver | moderate | T3N2M1 |
| Patient 4 | Female | 59 | liver | poorly differentiated | T3N2M1 |
| Patient 5 | Male | 62 | liver | poorly differentiated | T3N2M1 |
| Patient 6 | Male | 64 | liver | moderate | T3N1M1 |
| Patient 7 | Female | 75 | Rectum, liver | poorly differentiated | T3N2M1 |
| Patient 8 | Male | 78 | liver | poorly differentiated | T3N2M1 |
